# Synthesis and evaluation of MePEG-PCL diblock copolymers: surface properties and controlled release behavior

**DOI:** 10.1007/s40204-015-0040-4

**Published:** 2015-08-20

**Authors:** Anjan Kumar Mohanty, Utpal Jana, Prabal Kumar Manna, Guru Prasad Mohanta

**Affiliations:** Department of Pharmacy, Annamalai University, Annamalai Nagar, Chidambaram, Tamilnadu 608002 India

**Keywords:** Biodegradable polymers, Drug carrier, Micelle, CMC, EPR effect

## Abstract

The amphiphilic block copolymers are composed of various combinations of hydrophilic and hydrophobic block unimers. The variation in unimer ratio alters the surface as well as micelle-forming properties of the block copolymers. These nanoscopic micelles have the ability to encapsulate hydrophobic compounds and act as potential drug carrier. MePEG-PCL copolymers with various block lengths were synthesized by ring-opening polymerization and characterized by ^1^HNMR, GPC, WXRD and DSC. The number average molecular weight of the block copolymer was found to vary from 7511 to 21,270 as determined by GPC and ^1^HNMR studies. The surface topology of the polymer films was determined by AFM analysis, which shows a smoother surface with increased MePEG contents in the block copolymers. The protein-binding assay indicates a better biocompatibility of the block copolymers in comparison to MePEG or PCL alone. The CMC of the block copolymer provides the information about micelle formations for encapsulation of hydrophobic materials and affects the in vitro release.

## Introduction

Biodegradable block copolymers are well-recognized biomaterials for their biomedical applications. These copolymers are used for medical devices, along with their application in carry and release of drugs, peptides or proteins at the characteristic rates and specific target site (Saito et al. 2001; Uhrich et al. 1999). The block copolymers have the capability of self assembly into intermittent geometry with long-range order and contain at least two distinct polymer chains, covalently bound at one point. The copolymers have the ability to control their amphiphilic behavior, mechanical and physical properties by adjusting the ratio of the constituting blocks or adding new blocks of desired properties (Leenslag et al. 1987). The brisk development of block copolymers towards the drug delivery formulations is due to the versatile and flexible structural design. On functionalizing both the hydrophilic and hydrophobic part, the molecular weight of the block copolymers can be varied within a wide range while maintaining a constant hydrophilic–lipophilic balance (HLB). Moreover, the block copolymers are largely used due to low immunogenicity, biodegradability and biocompatibility, which make them suitable for the safe human administration (Aliabadi and Lavasanifar 2006; Deming 2000; Mahmud et al. 2007).

The biomaterials such as poly(lactide) (PLA), poly(lactide-co-glycolide) (PLGA), poly(ethylene glycol) (PEG) allow the attachment of proteins and peptides by attaching functional groups into the polymer side chains, which make them suitable for the cell penetration and adhesion (Adams et al. 2003; Lavasanifar et al. 2001; Mohanty et al. 2010). The PEG has the property of hydrophilicity, nontoxicity and block copolymers consisting of PEG chain can easily form micelles in aqueous solution at room temperature and this property helps in drug delivery using the polymers as carrier system. The sizes of the PEG blocks directly affect the balance of hydrophobicity and hydrophilicity within a block copolymer which modifies the drug carrying efficacy (Cook et al. 1997). The polyester, for example, poly(ε-caprolactone) (PCL) exhibits good biodegradability and biocompatibility and is extensively used for controlled drug delivery and tissue engineering applications in several formulations. Its compatibility with a wide range of drugs enables uniform drug distribution in the formulation matrix and its long-term degradation facilitates drug release up to several days. The PCL can be prepared by ring-opening polymerization of ε-caprolactone (CL) activated by catalyst and micro-initiator. These polymers are used to prepare amphiphilic block copolymers in aqueous environment with hydrophobic core by self assembling of lipophilic parts and vice versa (Jenkins and Harrison 2006; Pulkkinena et al. 2009). These copolymers have the advantage that their degradation does not result in an acid environment, unlike the degradation of PLA and PLGA (Kim et al. 2004). However, due to the high degree of crystallinity and hydrophobicity, PCL degrades rather slowly and less biocompatible with soft tissue, which restricts its further clinical application. Furthermore, to raise the undesirable long-term degradation of PCL, modifications in the form of blend or copolymers are done with other hydrophilic polymers (Burke et al. 2004).

The common approach to obtain biodegradable polymers that allow surface modification is the copolymerization of the PCL with PEG, which improves biodegradability as compared to the PEG alone The copolymers also show higher hydrophilicity, mechanical properties and better performance in the cell culture studies than the MePEG or PCL homopolymer (Huang et al. 2004; Moon et al. 2002). The hydrophilic PEG chains control protein and peptide adsorption and consequently regulate the behavior of cells to the polymer surface. In addition, the hydrophobic or hydrophilic core allows repositioning of encapsulated drug into the core, hence improves the solubility in a monophasic non-solvent medium. Despite many reports on the synthesis of PEG- and PCL-derived copolymers (Wang and Qiu 1993; Wang et al. 2013) and their potential medical and pharmaceutical applications (Koenig and Huang 1995; Wang et al. 2012), there is little detailed information available on the bulk microstructure and surface properties (Zhou et al. 2003; Adams et al. 2003) depending on the copolymer composition. However, the lipophilic drugs are generally distributed uniformly in the matrix, while the hydrophilic drugs tend to move towards the interface and remain on the surface of PEG-PCL formulation in the adsorbed state (von Burkersroda et al. 1997). From PEG-PCL-based investigations, it can be concluded that diffusion is the only possible mechanism by which the lipophilic drugs release from PEG-PCL formulations as they were reported to be intact for a much longer duration of in vivo application (Yang et al. 2014; Gou et al. 2009). In case of highly lipophilic drug resisting complete diffusion, the drugs are released upon surface erosion by enzymatic action. However, hydrophilic drugs that accumulate at the interface during the formulation processes are released by desorption at the initial period of release study or dosage intake. Thus, PEG-PCL block copolymer formulations release the drugs in a biphasic pattern where the burst release is much higher for hydrophilic drugs than lipophilic ones (Bazile et al. 1995; Dee et al. 1998; Wang et al. 2014).

We considered the properties of methoxypoly(ethylene glycol)-poly(ε-caprolactone) diblock copolymers (MePEG-PCL) in terms of surface topography along with polymer bulk microstructure to evaluate the contribution of the MePEG as well as the PCL part to the overall properties of the block copolymer. The model protein belonging to cell-penetrating peptides (amino acid composition) category was chosen, to check the covalent attachment of copolymer to cell proteins. The hydrolytic degradation and drug release results are intended to contribute to a better understanding of the surface properties of these promising biomaterials and may lead to their efficient use as drug and cell carriers in future applications.

## Materials and methods

### Materials

Methoxypoly (ethylene glycol) (MePEG, *M*_n_ = 5000 by supplier, *M*_n_ = 5244 by our GPC measurements) was supplied by Fluka (Sigma-Aldrich, St. Louis, MO), and purified by azeotropic distillation with dry toluene (Ranbaxy Fine Chem., India), and dried to constant weight under vacuum at 30 °C before use. ε-caprolactone (ε-CL) (*M*_w_ = 114.14) was purchased from Fluka and was dried over calcium hydride for 48 h at room temperature and then distilled under reduced pressure prior to polymerization. Stannous-II octoate (Sn(Oct)_2_), Pyrene, Trypsin from bovine pancreas, *N*α-benzoyl-l-arginine ethyl ester hydrochloride (BAEE), trypsin inhibitor from soybean, Tris(hydroxymethyl)-amino methane (TRIS) was obtained from Sigma-Aldrich Co. (Saint Louis, MO). Distilled-deionized water was prepared with Milli-Q plus System (Elix 10, Millipore Corp. India). All other chemicals used were of reagent grade and used as purchased without further purification.

### Polymer synthesis

MePEG (molecular weight, 5000 Da) was subjected to azeotropic distillation with toluene for 6 h to remove the entrapped moisture and excess toluene was removed by rotavapor. ε-CL was activated by keeping it in activated molecular sieves for 24 h. MePEG-PCL diblock copolymers were synthesized by a ring-opening polymerization of ε-CL using MePEG homopolymer as micro-initiator and Sn(Oct)_2_ as catalyst. Briefly, 5 mg of different ratios of MePEG and ε-CL was mixed in a 10-ml round bottom flask connected to vacuum. The mixture was degassed with vacuum pump (0.28 mbar) and kept at 160 °C. When the polymerization was completed, the reaction product was cooled at an ambient temperature, and precipitated. The precipitate was collected by filtration and washed several times with diethyl ether to remove any residual caprolactone unimers. The resulting product was dried in a vacuum oven at 40 °C for 3 days (Zhou et al. 2003). All block copolymers (MePEG20-PCL80, MePEG40-PCL60, MePEG60-PCL40, MePEG80-PCL20) with different MePEG and PCL ratios were synthesized by adopting the above method by keeping the final weight of the mixture constant at 5 mg.

### Determination of polymer molecular weight by GPC

The molecular weight and molecular weight distribution were determined by gel permeation chromatography (GPC). The chromatography was carried out at 35 °C temperature using HPLC system (Shimadzu Deutschland GmbH, Duisburg, Germany) fitted with LC-10AD HPLC pump and RID-6A refractive index detector coupled with a styragel HR4 column in an isocratic mode with the deaerated chloroform as mobile phase at a flow rate of 1 ml/min. The samples were prepared by dissolving 10 mg of polymer in 2 ml chloroform and filtered through 0.2 µm membrane filter (Millipore). 50 µl of the sample was injected and analyzed using “GPC for Class-Vp” software (Shimadzu Deutschland GmbH, Duisburg, Germany). The molecular weights were calculated from the elution volume of polystyrene standards with molecular weight range of 5610–275,000 Da (Polysciences Asia Pacific, Inc. Taiwan).

### ^1^H-NMR spectra

10–20 mg of each sample of copolymers was dissolved in CDCl_3_ and ^1^H-NMR spectra were taken at 300 MHz using Bruker (Avance, DPX 300, Germany). TMS contained in CDCl_3_ served as shift reference. The number average molecular weight of copolymer and PEG to PCL ratio was determined by integrating the signal pertaining to each molecule of the sample.

### Analysis of polymer bulk microstructure with WAXD

The wide-angle X-ray diffraction (WAXD) studies were carried out to know the crystallographic state of different MePEG-PCL diblock copolymers. The XRD patterns of the samples were obtained using XRD diffractometry (D8 ADVANCE, Bruker AXS Inc, Madison, WI). A monochromator features high flux *K* − *α* − 1 radiation at 40 kV and 100 mA was used. Diffractograms were performed with 2*θ* range from 0° to 40° with a step of 0.02°, at a scanning speed of 4°/min (2*θ*).

### Analysis of polymer bulk microstructure using DSC

The structures of the polymer bulk, the melting point temperature of polymers were characterized using a differential scanning calorimetric (DSC) thermogram analyser (STA 6000 Simultaneous Thermal Analyser, Perkin Elmer, Waltham, MA). To perform the DSC analysis, 5 mg of MePEG-PCL diblock copolymer samples was sealed separately in a standard aluminum pan, and purged with pure dry nitrogen gas set at a flow rate of 10 ml/min, the temperature variation was set at 10 °C/min, and the heat flow was recorded from 30 to 200 °C.

### Manufacture of MePEG-PCL diblock copolymer films

The polymeric surface properties were investigated by making thin film of MePEG-PCL diblock copolymers. 50 mg of polymers was dissolved in 1 ml of methylene chloride and 100 µl of the solution was spin-cast on glass slides to make uniform (~1 cm^2^ area) layer of film. The films were allowed to dry under laminar air flow (Esco laminar flow hood) for 2 h followed by at least 24-h drying under vacuum at room temperature in a desiccator to remove the residual solvent. Further, for complete removal of organic solvent, the samples were kept for 24 h under vacuum at 60 °C.

### Determination of polymer surface topography with AFM

The surface characteristics of the MePEG-PCL diblock copolymers film were investigated by atomic force microscopy (AFM) (JPK nanowizard II, JPK instrument, Berlin, Germany) mounted with Zeiss lens. The samples were scanned by intermittent air contact mode using pyramidal cantilevers with silicon probes having force constants of 0.2 Nm^−1^. The surface structure was observed and imaged using JPK data processing software in contact mode with frequency of 200–400 kHz and scan speed of 2 Hz. Under the chosen conditions, the observed features were stable in all four scanning directions as well as during multiple scans and at varying scan sizes. Cantilevers and parameters were proved for proper work by scanning the surface of a cover slide. All the images represent topographic data which were obtained from z-range amplification scanner controlled by the z-piezo extension scanner feedback loop at a gain of 0.25–0.1 arbitrary units. The raw data were reprocessed with the JPK-SPM data processing software (version spm-3.4.11+).

### Hydrolytic degradation of polymer films

The uniform and thin films (20 × 20 mm) of block copolymers were prepared by dissolving 20 mg of each sample in solvent and spin-casting the polymer solution followed by air drying. The hydrolytic degradation of the MePEG-PCL block copolymers was carried out in a 1 N NaOH solution at 37 °C. The samples and solution were incubated in a beaker and put in a water bath with gentle shaking. The samples were collected at predetermined time intervals, washed with distilled water and dried to a constant weight in a vacuum oven at 60 °C for weighing.

### Attachment of protein to polymer surfaces

The immobilized bovine trypsin was used as model substance, and was quantified according to its enzymatic reactivity (Puleo 1996; Tessmar et al. 2003). The MePEG-PCL diblock copolymers films were used for coupling reaction with trypsin in 0.05 m TRIS buffer (pH = 8.0). Trypsin was finally immobilized from a concentrated 1 mg/ml solution in TRIS buffer. The polymer films were incubated separately with 2 ml of this solution for 2 h. The polymer films were washed three times with PBS buffer (pH = 7.4 containing 0.05 % Tween 20) to remove excessive physically adhered trypsin. To determine the amount of immobilized protein, the polymer films were incubated with 2 ml of a 0.4 mg/ml solution of BAEE in TRIS buffer at 37 °C in 12-well plate (Corning, USA). After 2 h, the enzymatic reaction was stopped by adding 500 ml of a 0.02 mg/ml solution of soybean trypsin inhibitor in double distilled water. The UV absorbance was read at 253 nm using spectrophotometer (SPECORD^®^ 210, Analytik jena, Germany). The trypsin activity of polymer films was measured independently five times. Experimental values calculated from the absorption of sample solutions were corrected for UV absorption of BAEE blank solution.

### Critical micelle concentration (CMC)

The CMC of the micelles was measured using the fluorescence dye solubilization method. The hydrophobic fluorescent dye pyrene undergoes well-known changes in response to microenvironment polarity, so it was used as probe in a fluorescence spectrophotometer (LS55, Perkin Elmer, Waltham, USA). A 0.6 mM of pyrene in acetone solution was added to glass vial, 10 µl of this solution was mixed with 1.0 ml of the MePEG-PCL diblock copolymer aqueous solution with concentration ranging from 2.5 × 10^−3^ to 5.0 mg/ml and kept in an incubator overnight for equilibration (Arimura et al. 2005; Li et al. 2010). The excitation spectra were scanned from 250 to 360 nm at a fixed emission wavelength of 390 nm with bandwidth 3 nm. A sharp increase in the ratio of intensity at 338 and 333 nm (I_338_/I_333_) excitation occurs at the CMC as the pyrene preferentially partitioned into the hydrophobic cores of the micelles. The ratios of I_338_/I_333_ were calculated and plotted against the concentration logarithm of micelles; the CMC was obtained from the intersection of the two tangent plots (Lavasanifar et al. 2001; Winnik and Regismond 1996).

## Preparation of pyrene-loaded micelles and in vitro release kinetics

Pyrene-loaded MePEG-PCL diblock copolymeric micelles were prepared by phase-separation/dialysis method (Mohanty et al. 2010). Briefly, 100 mg of MePEG-PCL diblock copolymer (MePEG20-PCL80 or MePEG40-PCL60 or MePEG60-PCL40 or MePEG80-PCL20) and 20 mg of pyrene were dissolved in 10 ml DMF. To the above solution, 20 ml of water was added to induce micellization under agitation. The aqueous solution was placed in a dialysis bag (molecular weight cutoff: 12 kDa, average diameter 21 mm and average flat width 35 mm, as supplied, Sigma) and dialyzed against doubly distilled water for 36 h under shielded light to remove DMF. The resulted micelle solution containing pyrene was lyophilized for 24 h (−80 °C and <10 mm mercury pressure, Freezone 6lt, Labconco Corp., MO) using 1 % sucrose as cryoprotectant so that the micelles can easily re-disperse, when required for further in vitro and in vivo evaluations. The obtained dry powder was stored in a desiccator at 4 °C. The entrapment efficiency of micelles was determined by dissolving 10 mg of the lyophilized sample in 5 ml of acetone, sonicated for 2 min in an ice bath (VC 505, Vibracell Sonics, Newton, USA) and centrifuged at 13,800 rpm for 10 min at 25 °C (Sigma 1–15 K, Osterode, Germany) to get clear supernatant, which was then analyzed by fluorescence spectrophotometer (LS55, Perkin Elmer, Waltham, USA) (Mohanty et al. 2010). The entrapment efficiency of micelle was calculated according to the following equation:

$$ {\text{EE}} \, \left( {\% \,   {\raise0.7ex\hbox{$w$} \!\mathord{\left/ {\vphantom {w w}}\right.\kern-0pt} \!\lower0.7ex\hbox{$w$}}} \right)\; = \; \frac{\text{Weight of pyrene in micelle}}{\text{Weight of pyrene added }} \; \times \;100 $$

In vitro release kinetics of all the four different pyrene-loaded micelles were carried out separately by dispersing 50 mg of the pyrene-loaded micelles in 100 ml of PBS (0.1 M, pH 7.4, containing 0.1 % v/v of Tween 80). Tween 80 was used to maintain the sink condition (Aso et al. 1994). The micelle suspension was kept in Orbit shaker rotating at 150 rpm in 37 °C under shielded light. At predetermined time intervals, 2.0 mL of the sample was extracted and centrifuged at 13,800 rpm, 4 °C for 10 min, the supernatants were collected and measured by fluorescence spectrophotometer (LS55, Perkin Elmer, Waltham, USA); all measurements were performed in triplicates. After being measured, the supernatant and precipitant were immediately poured back for further analysis (Zhou et al. 2003).

## Results and discussion

### Polymer synthesis and polymer structure

**Fig. 1:**
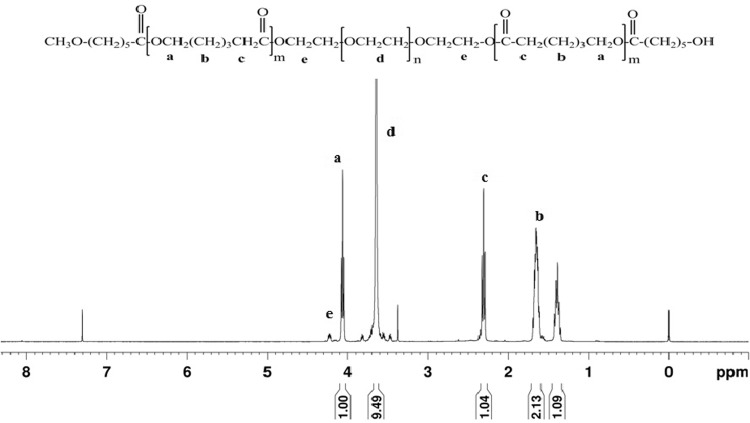
^1^H-NMR spectrum (300 MHz) of MePEG60-PCL40 in CDCl_3_

The synthesis of MePEG-PCL block copolymers was carried out by ring-opening copolymerization mechanism in presence of catalyst. The combination of MePEG and ε-CL by covalent bonding yields diblock copolymers with bulk microstructure containing MePEG hydrophilic part isolated from PCL hydrophobic part. The PCL hydrophobic domain contributes for long in vivo degradation times. The MePEG content varies in different formulation. The characterization of the block copolymers with the ^1^H-NMR shows the spectrum of MePEG which is significant for all synthesized MePEG-PCL diblock copolymers. The signal at 3.64 and 4.06 ppm is representative for three chemically equivalent hydrogen atoms of the methyl group at the end of the MePEG block and ε-methylene proton in PCL block in the polymer chains, respectively (Fig. 1). The GPC analysis of polymer shows molecular weight relative to the poly(styrene) standards used for the calibration of the system. The polydispersity indices of the MePEG-PCL diblock copolymers indicate a narrow molecular weight distribution for all polymers (Table 1). These indicate that no transesterification and/or backbiting reactions occurred during the copolymerization. The experimentally determined block length of the MePEG chains has some deviation from the manufacturer declaration; however, the discrepancy was less than 5 % and it confirms that polymers can be synthesized consistently (Peter et al. 1997).

**Table 1:** Molecular weight and composition of MePEG-PCL copolymers

Sample	Feed molar ratio (PCL/MePEG)^a^	*M*_n_ (Calc.)^b^	*M*_n_ (Expt.)^a^	*M*_w_ (Expt.)^a^	PDI	Composition weight (%) (MePEG/PCL)^c^
MePEG100-PCL0	0	5000	5244	5873	1.12	100:0
MePEG20-PCL80	175	25,000	21,270	28,714	1.35	24:76
MePEG40-PCL60	65.75	12,500	12,213	16,853	1.38	42:58
MePEG60-PCL40	29.16	8333.3	7511	9313	1.24	65:35
MePEG80-PCL20	10.96	6250	5872	7927	1.35	82:18

All molecular weight data were rounded to the nearest 100

^a^Determined on the basis of *M*_n_ of polymer calculated in GPC experiments and polydispersity indices (PI = *M*_w_/*M*_n_)

^b^Calculated from MePEG (*M*_w_ = 5000)

^c^Determined by ^1^HNMR spectroscopy (CDCl_3_)

### Analysis of polymer bulk microstructure with WAXD

**Fig. 2:**
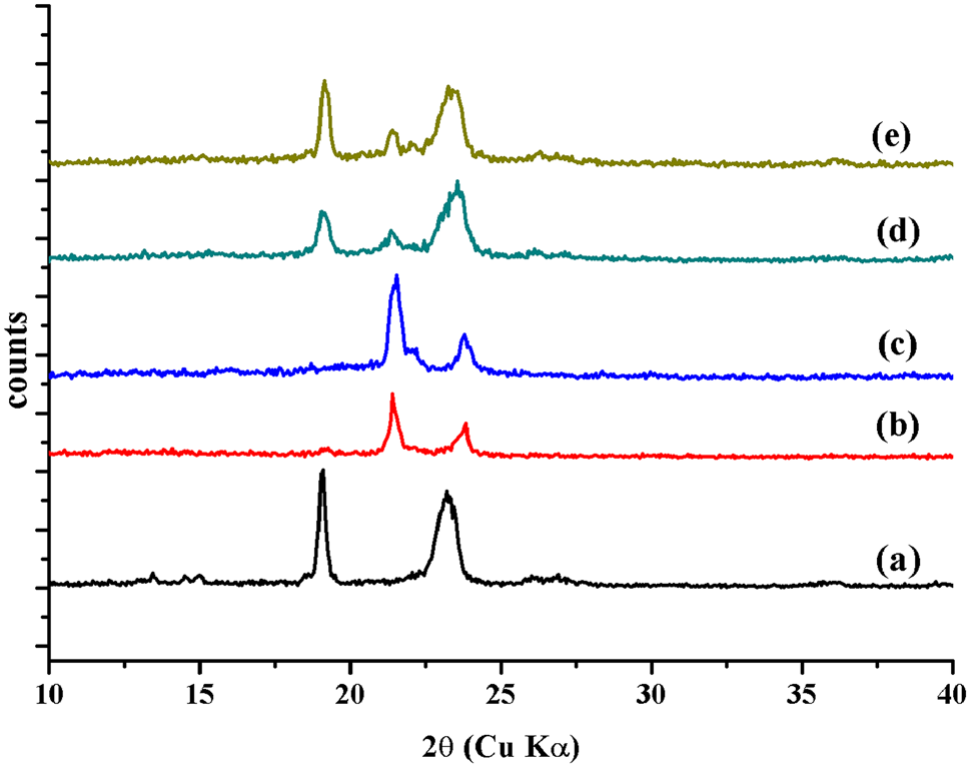
WAXD spectra of *a* PEG, *b* MePEG20-PCL80, *c* MePEG40-PCL60, *d* MePEG60-PCL40, *e* MePEG80-PCL20

The wide-angle X-ray diffraction (WAXD) analysis of MePEG polymer and MePEG-PCL diblock copolymers are carried out. The diffractograms of MePEG show a sharp diffraction maxima at 2*θ* = 19º, 23º and 28º. It is well known that the PCL homopolymer is easy to crystallize. The diffraction patterns of the diblock copolymers show a broadly distributed signal in the range of 2*θ* = 10º–25º, which are related to the peaks of both MePEG and PCL block (Fig. 2). It demonstrated that the conjugation of MePEG and PCL suppressed the crystallization of PCL to desirable extent and both the polymer chains are mixed well in molecular level. In the diffractogram of MePEG20-PCL80, the diffraction decreases due to lower content of MePEG (Detchprohm et al. 2001; Senda et al. 2002). Moreover, the presence of the sharp diffraction maxima and halo in the WAXD diffractograms of MePEG-PCL block copolymers represents crystalline and amorphous polymer parts, respectively.

### Analysis of polymer bulk microstructure using DSC

**Fig. 3:**
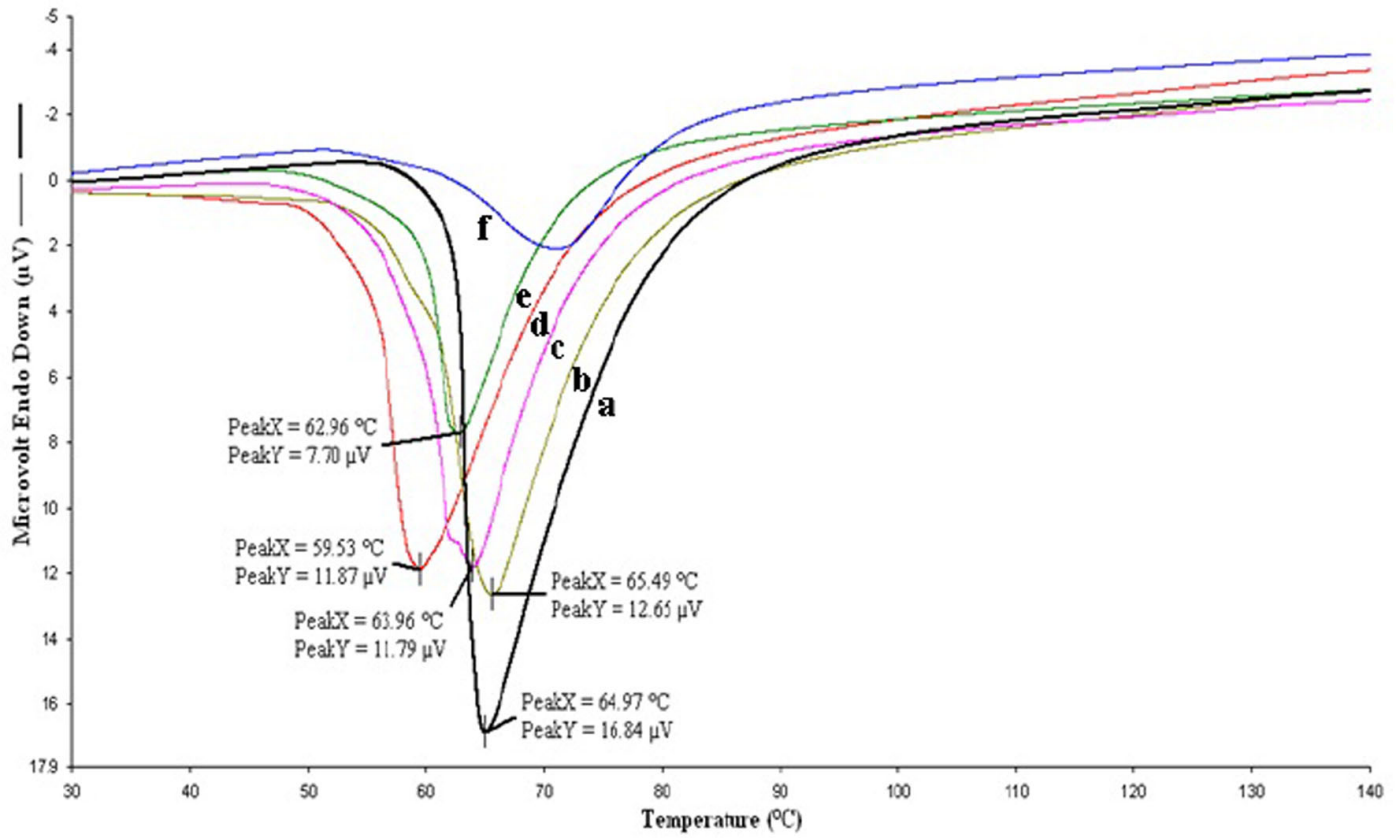
Thermograms obtained by DSC measurements for determination of the crystallinity of the polymers *a* MePEG, *b* MePEG20-PCL80, *c* MePEG40-PCL60, *d* MePEG60-PCL40, *e* MePEG80-PCL20, *f* PCL

The DSC analysis allowed the quantitative evaluation of thermal properties of the polymers such as melting point. The thermograms of MePEG included a single, symmetrical endotherm peak at 64 °C. PCL has well-known semi-crystalline polymer and thermograms of MePEG-PCL diblock copolymers had endotherm peaks at 65, 63, 62 and 59 °C for MePEG20-PCL80, MePEG40-PCL60, MePEG60-PCL40 and MePEG80-PCL20, respectively. The single endotherms in the thermograms are the result of the melting of both the PCL and MePEG blocks (Fig. 3). However, as the MePEG content within the copolymer decreases, the endotherm peaks for the MePEG-PCL block copolymers approach the peak of PCL homopolymer (Allen et al. 1999; Skoglund and Fransson 1996). The shift in melting point to lower temperatures demonstrates that there are interactions between both polymer chains, due to their covalent attachment which limits the mobility of MePEG and PCL chains. The chain lengths of MePEG and PCL influence the size and distribution of the polymer chains, thus determining the polymer bulk microstructure. The difference in hydrophilic part will most likely result in difference in water uptake which ultimately influences the degradation rate of polymers and release of incorporated drug (Aso et al. 1994; Rashkov et al. 1996; Youxin et al. 1994).

### Determination of polymer surface topography with AFM

**Fig. 4:**
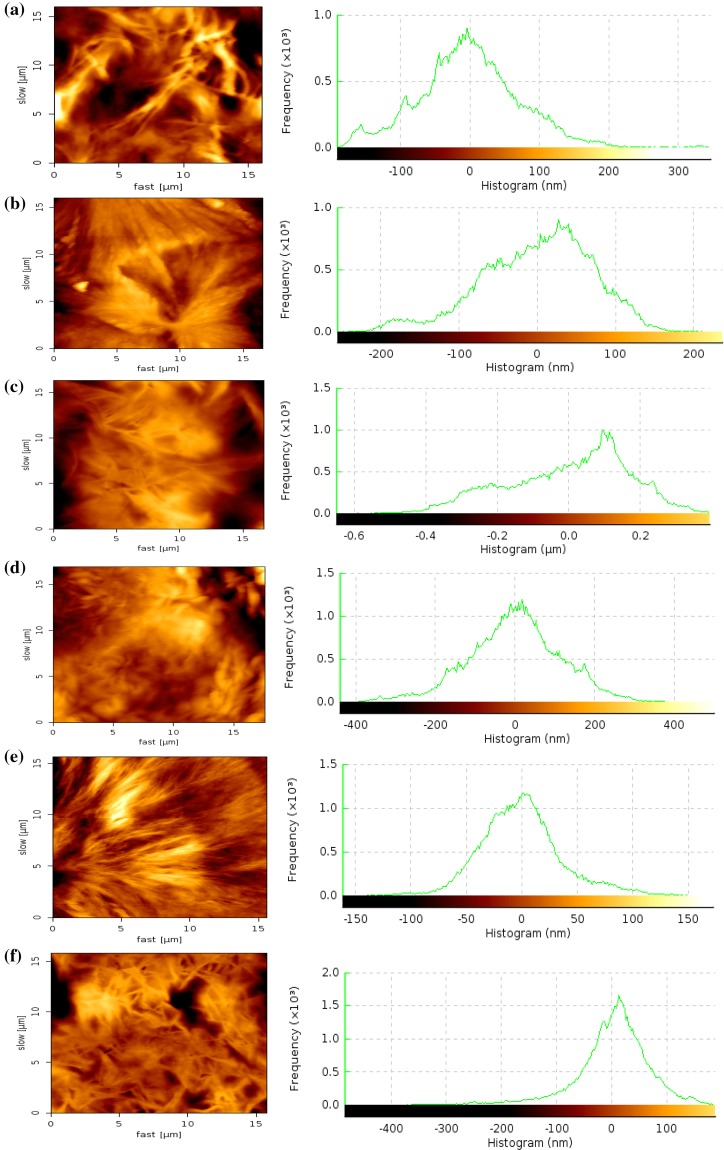
Surface topography of polymer films by AFM studies **a** PCL, **b** MePEG20-PCL80, **c** MePEG40-PCL60, **d** MePEG60-PCL40, **e** MePEG80-PCL20, **f** MePEG

The bulk microstructures of the MePEG-PCL block copolymers also influence the surface properties. The AFM analysis of MePEG, PCL and different MePEG-PCL block copolymers was carried out to get the two-dimensional plots and histogram of surface topographies (Fig. 4). The microphase separation pattern of this material, which was mostly pronounced in the phase image, was characterized (Lucke et al. 2000; Xiong et al. 1995). The plots were shown for different MePEG-PCL block copolymers with varying MePEG content and for pure PCL. The images of MePEG-PCL block copolymer films and histogram show small elevations on their surface, however, smooth polymer surface topography formed by increased MePEG content (Lucke et al. 2000; Xiong et al. 1995). The phase contrast was related to the fact that at room temperature, PCL was in a glassy state while MePEG was in a rubber-like state. Consequently, the brighter areas in the phase image (corresponding higher areas in topographic image) can be attributed to stiff lamellae of PCL. With increase in MePEG content of the polymers, the smoothness of the surface increases and the surface topology of MePEG had smooth topology with uniform histogram.

### Hydrolytic degradation of polymer films

**Fig. 5:**
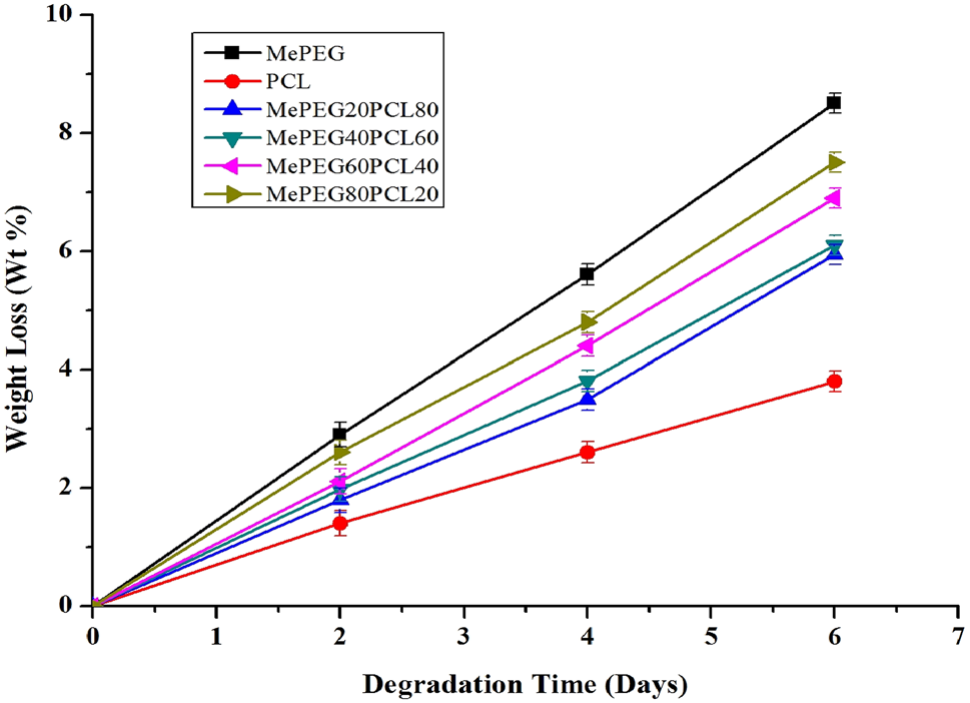
The weight loss of block copolymers as a function of hydrolysis time in the 1 N NaOH solution

The hydrolytic degradation occurs more prominently in amorphous substances as compared to crystalline substances. The higher degradation rate in the amorphous region is attributed to the easy diffusion of water molecules into the interior of polymers. Figure 5 shows the weight loss of polymer samples during hydrolytic degradation. The degradation rate of block copolymers was slower than those of MePEG depending on the crystallinity of the block copolymers (Cho et al. 2001). The results show that the hydrolytic degradation takes place preferentially in the amorphous region rather than in the crystalline region (Tsuji and Ikada 1997).

### Determination of protein attachment to polymer surfaces

**Fig. 6:**
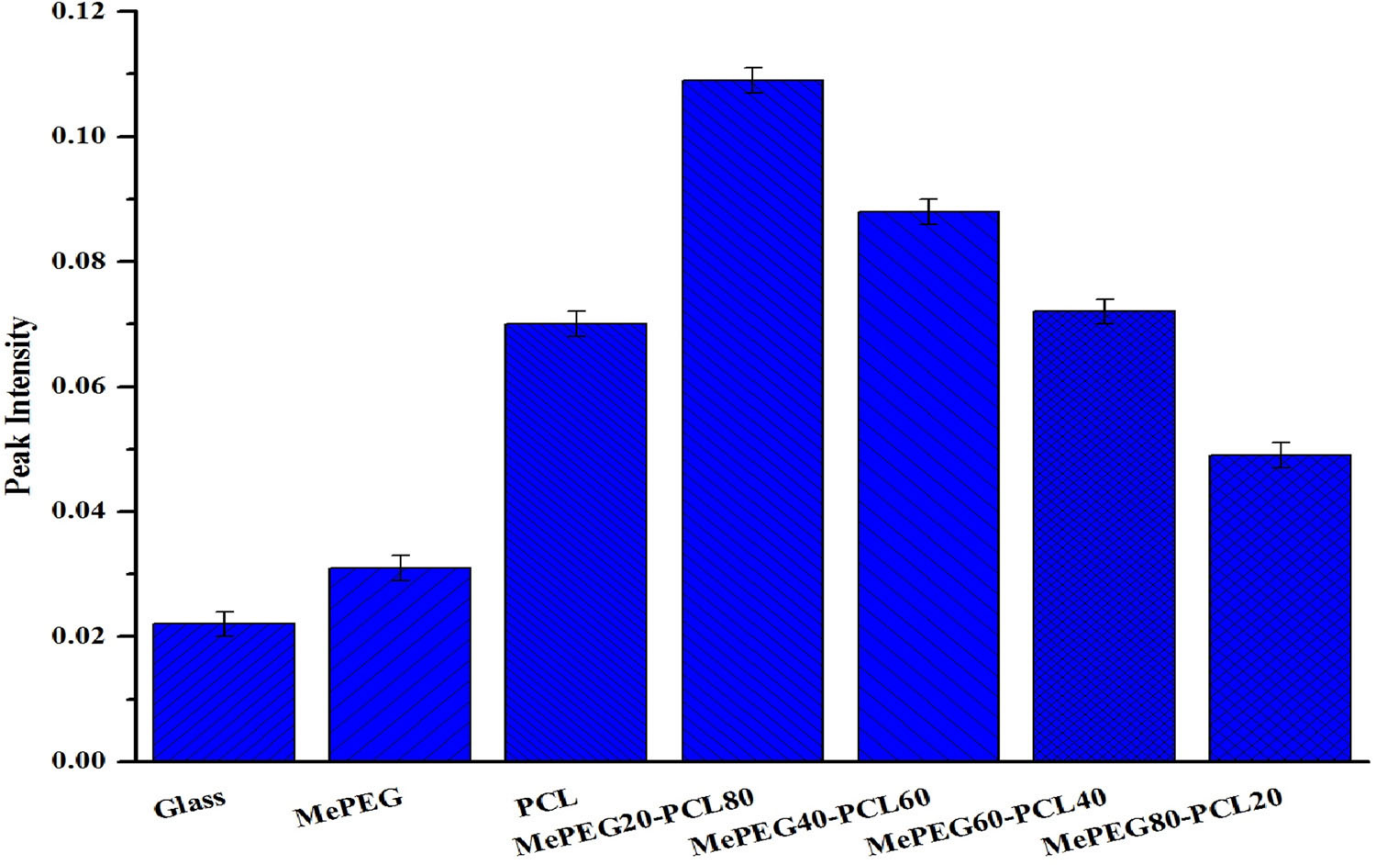
Binding of trypsin to different surfaces, determined by BAEE assay (UV detection *λ* = 253 nm)

The polymer surface biocompatibility and ability to form covalent bond with proteins and peptides was investigated by taking trypsin as a model protein. Different polymers (MePEG-PCL block copolymers, MePEG and PCL) and the non-coated glass slides were studied and enzymatic activity was calculated (Fig. 6). All the experiments were performed three times and the average enzymatic activity was calculated (Puleo 1996; Tessmar et al. 2003). The UV absorption measures showed that MePEG20-PCL80 block copolymer films had higher absorption than other polymer films, due to presence of MePEG on the polymer surface (Andrade et al. 1992; Bazile et al. 1995; Stolnik et al. 1994). The sensitivity of UV-spectroscopic method was illustrated by small standard deviations when Nα-benzoyl-l-arginine (BA) was cleaved from Nα-benzoyl-l-arginine ethylester (BAEE). The UV absorbance for glass and PCL that served as negative controls was relatively high, due to adsorption of protein on the bottom of the glass slides. The increases in UV absorption show the ability of MePEG-PCL block copolymers to covalently bind with immobilize trypsin (Puleo 1996; Tessmar et al. 2003). However, the results verify that the four block copolymers have different adsorption properties depending on the MePEG chains, and it was suited to bind bioactive substances in solid phase (Lucke et al. 2000). The MePEG polymer surface minimizes the unspecific cell adhesion of polymer, and in the same time it helps in functionalization of polymer surface by covalent bonding of targeting moieties that mediate the selective target to specific tissues/cells by the enhanced permeability and retention (EPR) effect. The control of peptide and protein adsorption on the surface of polymer is essential to control cell function.

### Critical micelle concentration (CMC)

**Fig. 7:**
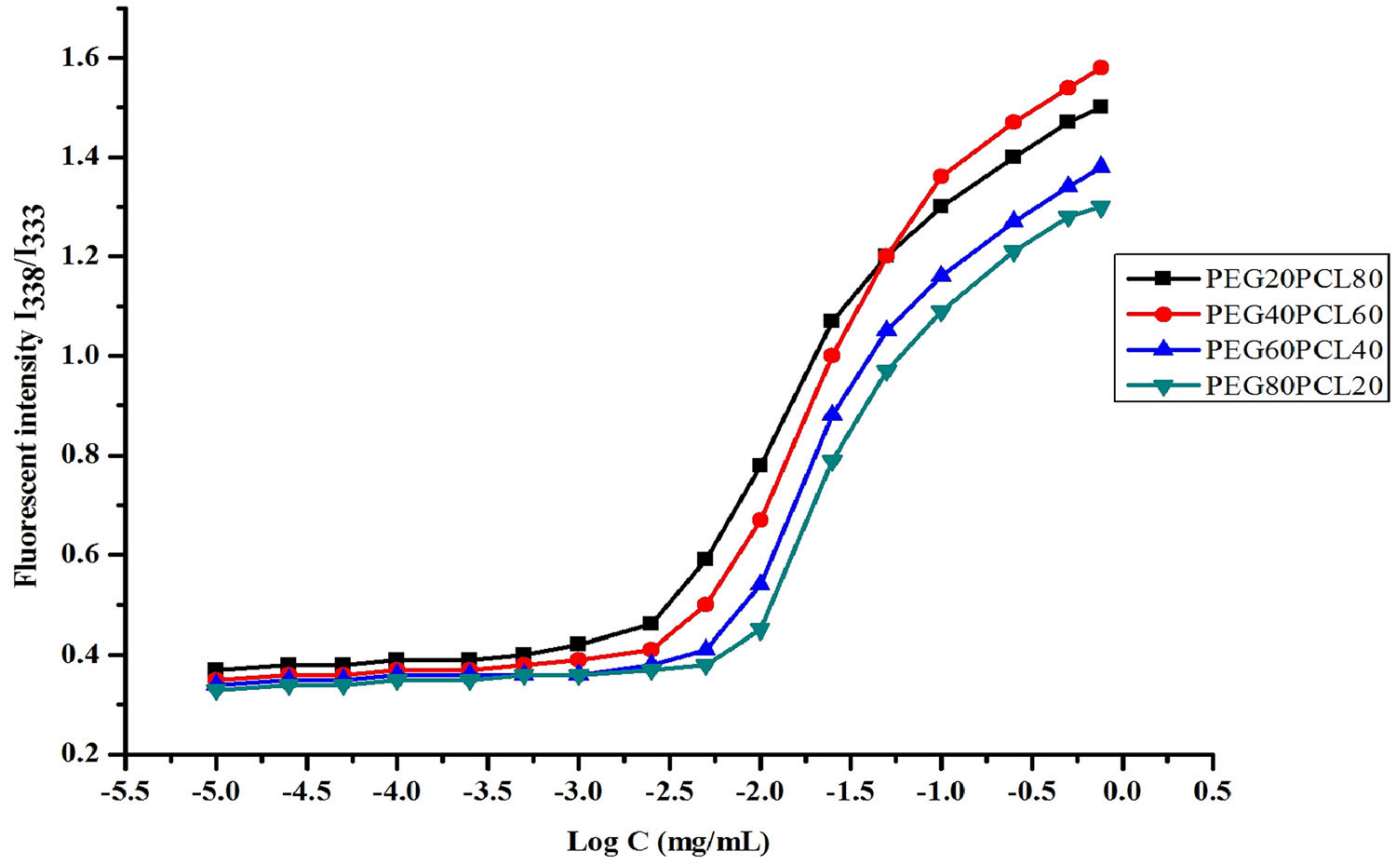
Plots of intensity ratio (I_338_/I_333_) of the excitation spectra of pyrene against log C of block copolymers

The CMC value of block copolymer indicates the stability of micellar formulation upon dilution in body fluids followed by intravenous administration. The formation of the micelles was evaluated by CMC measurement using pyrene as a florescent probe. Pyrene was preferentially partitioned into the hydrophobic domain of the micelles and to make change in polarity or photophysical properties of the solution. The excitation spectra of pyrene shift from 333 to 338 nm. The ratio of pyrene fluorescence intensities at 338 and 333 nm (I_338_/I_333_) was plotted as a function of logarithm of block copolymer concentration of all polymers. The CMC value of MePEG80-PCL20 (8.2 × 10^−3^ mg/ml) was higher than the CMC value of MePEG60-PCL40 (5.9 × 10^−3^ mg/ml), MePEG40-PCL60 (4.8 × 10^−3^ mg/ml) and MePEG20-PCL80 (3.9 × 10^−3^ mg/ml). The intensity ratio remained almost unchanged at low copolymer concentrations and increased abruptly once the copolymer concentration reached the CMC, indicating the formation of micelles (Kabanov et al. 1995; Piao et al. 2003). The CMC value depends on the hydrophilic–lipophilic balance (HLB) of block copolymers, and increases with increase in the hydrophilic block lengths, leading to instability of micelles (Xiong et al. 1995; Yokoyama 2010). The lower CMC of MePEG20-PCL80 and MePEG40-PCL60 indicated their higher thermodynamic stability, representing an advantage upon injection into body fluids. This was due to incorporation of PCL units to the block copolymer chain (Fig. 7).

## Preparation of pyrene-loaded micelles and in vitro release kinetics

**Fig. 8:**
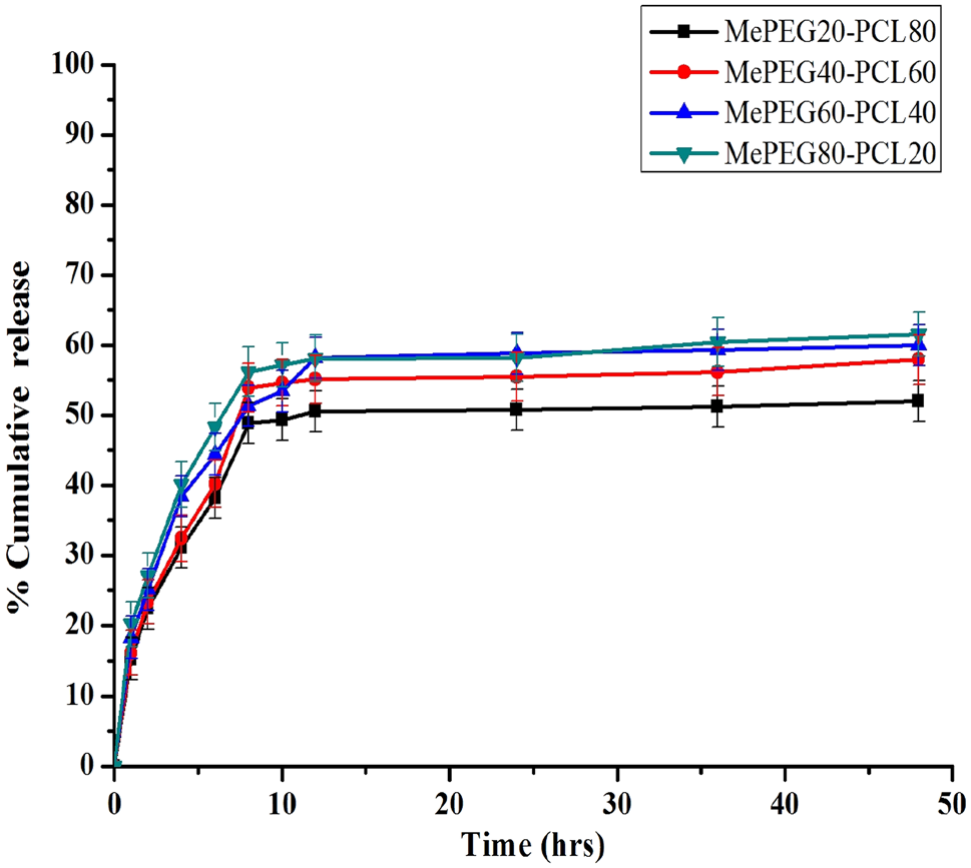
In vitro release kinetics of pyrene from different formulation of MePEG-PCL copolymeric micelles in PBS (0.01 M, pH 7.4) at 37 °C. (Data as mean ± standard error of mean, *n* = 3)

The pyrene was successfully loaded in the different MePEG-PCL block copolymeric micelles by modified dialysis method. The entrapment efficiency of different micellar formulation was an important factor for determining the release profile of drug delivery system. The entrapment efficiency of pyrene in different formulation was determined by fluorescence spectrophotometer and found to be ~57.2 % (MePEG80-PCL20), ~63.1 % (MePEG60-PCL40), ~67.4 % (MePEG40-PCL60) and ~70.1 % (MePEG20-PCL80). The higher molecular weight and presence of large hydrophobic chain length of block copolymer results in high entrapment of pyrene (Shin et al. 1998; Yokoyama 2010). The release profile of pyrene from the different MePEG-PCL micelles was carried out in vitro physiological body fluid condition (PBS 0.01 M, pH = 7.4, 0.1 % v/v Tween 80) for 48 h. The release of pyrene shows a typical biphasic pattern, indicating the first burst release due to rapid diffusion of pyrene from polymeric matrix and later phase release was mediated by degradation of the polymeric matrix (Fig. 8). In case of MePEG80-PCL20 and MePEG60-PCL40 micellar formulation, pyrene release was found to be 61 ± 1.3 % and 59 ± 1.6 %, respectively, in the first instance, followed by slow and continuous release. Similarly, in case of MePEG40-PCL60 and MePEG20-PCL80 micellar formulations, the release was 56 ± 1.4 and 51 ± 1.2 %, respectively, in the first instance followed by sustained release (Ma et al. 2008). It was reported that as loading of the drug in micelle increases, the release rate decreases (Kim et al. 2004; Shin et al. 1998). The higher entrapment efficiency of MePEG20-PCL80 micellar formulation increases the hydrophobic properties of pyrene in core, favors enhanced interaction between pyrene and core of micelle, leading to decreased drug release (Uhrich et al. 1999).

## Conclusions

A series of MePEG-PCL biodegradable block copolymers were synthesized and explored its application for drug delivery. The results obtained show that the bonding of various compositions of hydrophobic PCL and hydrophilic MePEG leads to the desired effect on polymer bulk microstructure, surface properties and hydrolytic degradation. The variation of polymer composition regulates the ability of binding of model substances, such as proteins in solution or in solid phase. The CMC of the block copolymer shows the micelle-forming ability of the MePEG-PCL block copolymers to encapsulate hydrophobic drug. The in vitro release kinetic studies showed that the release of encapsulated hydrophobic substance has initial burst release followed by sustained release. Theses specifically designed MePEG-PCL block copolymer may be further explored as vehicle for controlled drug delivery by escalating EPR effect. The modification on polymer properties can aid in the development of customized release profiles. These copolymers can also be further modified as biomimetic copolymers by exploiting covalently bonded growth factors and cell adhesion peptides, which can be used for controlled tissue engineering.
